# *Turtling*: a time-aware neural topic model on NIH grant data

**DOI:** 10.1093/bioadv/vbad096

**Published:** 2023-07-24

**Authors:** Ruiyi Zhang, Ziheng Duan, CheYu Lee, Dylan Riffle, Martin Renqiang Min, Jing Zhang

**Affiliations:** Department of Computer Science, University of California, Irvine, CA 92697, United States; Department of Computer Science, University of California, Irvine, CA 92697, United States; Department of Computer Science, University of California, Irvine, CA 92697, United States; Department of Computer Science, University of California, Irvine, CA 92697, United States; Department of Machine Learning, NEC Labs America, Princeton, NJ 08540, United States; Department of Computer Science, University of California, Irvine, CA 92697, United States

## Abstract

**Motivation:**

Recent initiatives for federal grant transparency allow direct knowledge extraction from large volumes of grant texts, serving as a powerful alternative to traditional surveys. However, its computational modeling is challenging as grants are usually multifaceted with constantly evolving topics.

**Results:**

We propose Turtling, a time-aware neural topic model with three unique characteristics. First, Turtling employs pretrained biomedical word embedding to extract research topics. Second, it leverages a probabilistic time-series model to allow smooth and coherent topic evolution. Lastly, Turtling leverages additional topic diversity loss and funding institute classification loss to improve topic quality and facilitate funding institute prediction. We apply *Turtling* on publicly available NIH grant text and show that it significantly outperforms other methods on topic quality metrics. We also demonstrate that *Turtling* can provide insights into research topic evolution by detecting topic trends across decades. In summary, *Turtling* may be a valuable tool for grant text analysis.

**Availability and implementation:**

*Turtling* is freely available as an open-source software at https://github.com/aicb-ZhangLabs/Turtling.

## 1 Introduction

Advances in machine learning algorithms and the recent initiatives for federal grant transparency have allowed direct knowledge extraction from large volumes of publicly available online databases, potentially serving as a powerful alternative to traditional survey-based technologies. As a result, it is now possible to directly obtain quantitative and less biased grant text information that can broadly benefit scientific investigators, policy analysts, and funding agencies. Here, we aim to comprehensively navigate the funding landscape by exploring 466 730 public grant texts over the past 36 years from the National Institute of Health (NIH), the world’s largest funding agency for biomedical research.

Computational modeling on NIH grant text data can be challenging for two reasons. First, NIH grant texts are usually multifaceted because they can be individually or jointly awarded from 27 distinct institutes/centers (ICs) with overlapping priorities. Second, research topics have evolved quickly over the past decades as new technologies or health challenges have appeared (e.g. HIV and COVID pandemics in the 1980s and 2020s).

Previous researchers have leveraged topic models on NIH grant text to discover patterns reflecting latent research topics (Talley *et al.* 2011). Topics learned from their methods are robustly correlated with specific NIH institutes, providing a basis for the discovery of interrelationships among biomedical concepts from NIH grant abstract documents. Later on, other researchers have used a labeled topic model to take the institute category information into consideration (Park *et al.* 2016). Their work showed how text classification techniques can be used to analyze funding patterns of a specific institute. However, two problems limited the application of their models. First, training NIH data from scratch cannot capture rare word distributions. Second, while research topics have changed dramatically over the past 20 years, authors there used a static model that cannot capture temporal evolution information of research topics. Recently, some new topic modeling methods have been developed to capture topic trends in the general NLP area (Blei *et al.* 2003, Blei and Lafferty 2006, Dieng *et al.* 2019, 2020). Specifically, they use pretrained word embeddings to improve their topic quality and probabilistic time series to allow topics to vary smoothly over time. Nevertheless, it is challenging to directly apply them to NIH grant data due to its rare biomedical terminologies and complicated institute category information.

To tackle these challenges, we propose *Turtling*, a time-aware neural topic model with multitask losses, which encourages diverse topics and IC classification. *Turtling* has three unique characteristics compared with existing models. First, *Turtling* extracts topics from biomedical word embedding space, lessening the word scarcity problem. Second, it leverages a probabilistic time-series model, which allows smooth and coherent topic evolution. Lastly, *Turtling* leverages additional topic diversity (TD) loss and IC classification loss to further improve extracted topic quality and topic correlation with specific NIH institutes. The losses above contribute to the extraction of diverse and high-quality topics that contain IC-specific information.

To verify its applicability, we have collected the *Grant* dataset, which includes 466 730 grant abstract documents and their corresponding ICs across 36 years (1985–2020). We tested the performance of *Turtling* against baseline methods on the extracted topic quality and IC prediction accuracy using *the Grant* dataset. Our experimental results showed that our method significantly outperformed baselines on topic coherence (TC), diversity, and perplexity. Furthermore, we used our model to detect the topic trend across decades, providing valuable information on the evolution of research interests in the biomedical field. We then leveraged the topic proportions of a grant to predict its best-suited IC for success. We also found that grants from the same IC share similar topics in our visualizations as their topic proportion vectors were closer to each other, allowing for more interpretable predictions of IC selection given the grant abstract. In summary, our method provides an unbiased way for retrieving meaningful topics in NIH grants and its relation with NIH ICs.

## 2 Methods

### 2.1 Dataset

**Figure 1.**
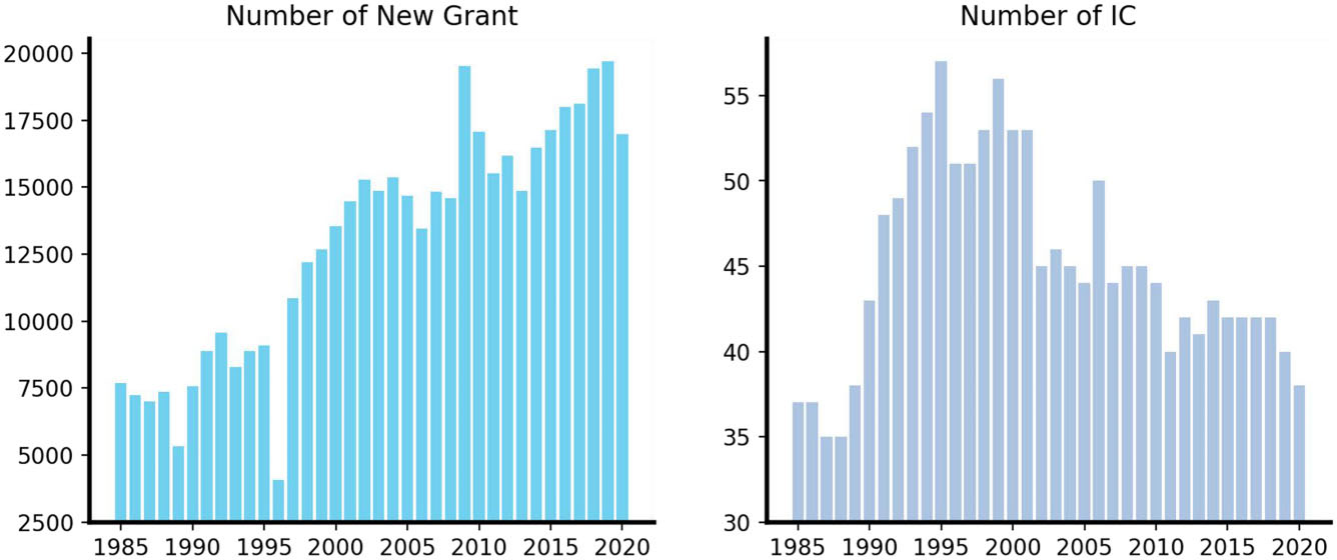
Statistics of the grant dataset. Left panel ss the number of new grants every year from 1985 to 2020, and right panel shows the number of ICs every year.

We collect 466 730 grant abstract documents from the NIH RePORTER website offered by the NIH to construct the *Grant* dataset (https://reporter.nih.gov/). We download the raw text data from the RePORTER website updated on July 26, 2022. The documents are across 36 years from 1985 to 2020. Each document is submitted to a certain IC. Figure 1 shows the number of new grants and new ICs every year. Among all ICs in our dataset, there are 62 that have been active for more than 10 years. As many grants receive funding for multiple years, we only include grants that received support for the first time.

We preprocess the *Grant* dataset by filtering out stop words and words with extremely high or low frequency. Specifically, we remove words that have a high frequency, appearing in more than 80% of a document, as well as words that have a frequency of less than 10 times in a document. We then use the Wordnet lemmatizer in NLTK to get the stem for each word (Bird and Loper 2004). After preprocessing, we further remove documents that contain less than 10 words. In total, we obtained a vocabulary with 35 108 distinct words.

### 2.2 *Turtling’s* topic modeling with word embeddings

**Figure 2.**
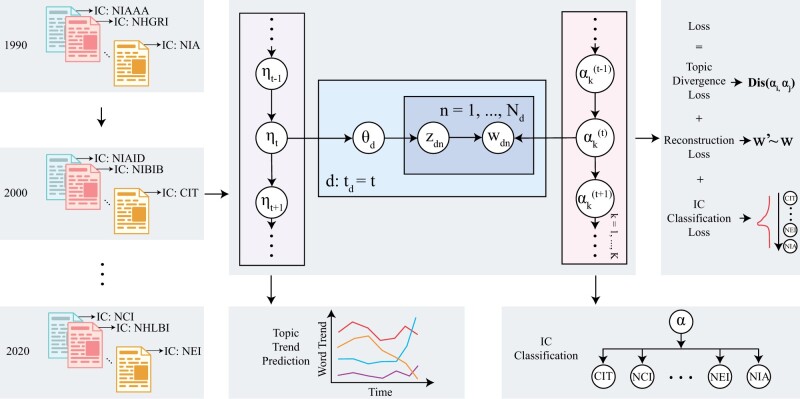
Flowchart of *Turtling*. *Turtling* leverages time-aware graphical topic model to extract high quality topics from grant documents across several years. The extracted topics can be used for several downstream tasks such as topic trend analysis and IC classification.

As shown in Fig. 2, *Turtling* adopts recent advances in probabilistic generative models of documents, such as latent Dirichlet allocation and word embeddings (Blei *et al.* 2003, Dieng *et al.* 2020). Specifically, *Turtling* leverages vectorized word embeddings to calculate the word distribution for each topic and assumes that the semantically related word embeddings and topic embeddings are closer to each other in the embedding space (Mikolov *et al.* 2013a,b).

As shown in Table 1, we use a vector $d_{tj}\in R^{V}$ to denote the bag of words (BOW) representation for the *j*th document in year t, where V is the size of the vocabulary and t represents a specific year. We then use $D_{t}\in R^{N_{t}\times V}\;$to denote the concatenation of all $N_{t}$ vectors $d_{tj}\;(1\leq j\leq N_{t})$, where $N_{t}$ is the number of grants for year t. Therefore, $D_{t}$ is a matrix that contains BOW information for all of the grant documents in year t. We then use $D_{t}=\{D_{1},D_{2},\ldots {,D}_{T}\}$ to denote our complete dataset, where *T* stands for the total number of years. For each BOW vector $d\in D_{t}$, we assign a corresponding label $y_{d}\in \{1,2,\ldots ,M_{t}\}$ to the document based on the IC it was submitted to. $M_{t}$ denotes the total number of ICs at a single year t.

**Table 1.** List of symbols^a^.

Symbol	Remark
$d_{tj}$	BOW vector for the *j*th document in year t
$D_{t}$	Document dataset at time *t*
$\theta_{d}$	Topic proportion of document *d*
$\beta_{k}$	Word distribution for topic *k*
$\alpha_{k}$	Embedding for topic *k*
$\eta_{t}$	Prior of topic proportion at time *t*
$z_{dn}$	Topic assignment for *n*th word in document *d*
$w_{dn}$	*n*th word in document *d*
Cat	Categorical distribution
LN	Logistic normal distribution

a We list the important symbols and notations used in this article and briefly describe each symbol.

We first consider the modeling process on a single year dataset. We define *K* topics $\beta_{i}(1\leq k\leq K)$, where each topic is a word distribution over the vocabulary, and *K* topic embeddings $\alpha_{k}(1\leq k\leq K)$ with the same dimension as word embeddings. The word embedding $\rho \in R^{L\times V}$ contains all of the words in the vocabulary, and *L* is the dimension of the embedding. We then calculate word distribution for each topic in Equation (1) as follows: where $\mathrm{Softmax}{\left(z\right)}_{i}=\frac{e^{z_{i}}}{\sum_{j}e^{z_{j}}}.\;$In this way, it calculates the generative probability for each word in proportion to the cosine similarity between each word embedding and the topic embedding. In the document generation process, we sample each word from its corresponding topic using this generative probability.


(1) βk=SoftmaxρTαk 1≤k≤K,

Then, we further consider a topic proportion vector $\theta_{d}$ with dimension *K* for each document, and each element of $\theta_{d}$ represents the probability of that topic to appear in document *d*. Formally, the generative process is as follows:

- Sample topic proportion $\theta_{d}∼\mathrm{LN}(0,I)$
- For *n*th word $w_{dn}$ in document *d*
  - Sample topic assignment $z_{dn}∼\mathrm{Cat}\left(\theta_{d}\right)\left(1\leq z_{dn}\leq K\right)$ Sample word $w_{dn}∼\mathrm{Cat}(\beta_{z_{dn}})$

where *LN* denotes the logistic normal distribution and Cat denotes the categorical distribution (Blei and Lafferty 2007). $z_{dn}$ is an integer that takes value from 1 to *K*.

### 2.3 Time-aware topic modeling

We then extend the method mentioned above to evolve dynamically on a multiyear dataset by allowing topics to vary smoothly over time. Within this model, the number of topics, denoted as *K*, remains consistent throughout all years, though the topic embeddings for each year exhibit slight variations compared to those from preceding years. Formally, for each time point *t*, *Turtling* defines a time specific topic embedding $\alpha_{k}^{t}\in R^{L}$. Similarly, it calculates the time-specific word distribution $\beta_{k}^{t}\in R^{V}$ for each topic with the following formula:


(2) βkt=SoftmaxρTαkt 1≤k≤K.

Different from the method in Section 2.2, the time-specific topic distribution for each document $\theta_{d}^{t}$ is generated from a distribution that also evolves over time: where ϵ is a hyperparameter of the model and $\eta_{t}$ is a latent variable that defines the prior mean of topic proportion at a specific time *t*. We assume that every $\eta_{t}$ is a vector with dimension *K* generated by a random walk starting from $\eta_{t-1}$ with Gaussian noise δ, so the conditional distribution of $\eta_{t}$ given $\eta_{t-1}$ is as follows:


(3) θdt∼LNηt,ϵ2I,

(4) pηtηt-1=LNηt-1,δ2I.

Similarly, we assume the topic representation also evolves by random walk with Gaussian noise γ:


(5) pαktαkt-1)=LNαkt-1,γ2I.

At time step t=0, we assume both $\alpha_{k}^{0}$ and $\eta_{0}$ follow Gaussian distribution N(0,I). Thus, the generative process of *Turtling* can be summarized as follows:

1. Sample initial topic embeddings $\alpha_{k}^{0}∼N(0,I)$
2. Sample initial topic proportion mean $\eta_{0}∼N(0,I)$
3. For time step *t* = 1, 2, …, *T*:
  - Sample topic embeddings $\alpha_{k}^{t}∼\mathrm{LN}(\alpha_{k}^{t-1},\gamma^{2}I)$
  - Sample topic proportion mean $\eta_{t}∼\mathrm{LN}(\eta_{t-1},\delta^{2}I)$
  - Calculate $\beta_{k}^{t}=\mathrm{Softmax}(\rho^{T}\alpha_{k}^{t})$
4. For each document $d\in D_{t}$:
  - Sample topic proportion $\theta_{d}∼\mathrm{LN}(\eta_{t},\epsilon I^{2})$
  - For each word, $w_{dn}$ in document *d*:
    - Sample topic assignment $z_{dn}∼\mathrm{Cat}(\theta_{d})$
    - Sample word $w_{dn}∼\mathrm{Cat}(\beta_{z_{dn}}^{t})$

Since *Turtling* learns topics in an embedded space, it can assign topics to words that do not appear in the training corpus as long as their embedding is given.

### 2.4 Inference of topic proportion and topic assignment

Given a word $w_{dn}$ in document *d* at time *t*, we then calculate the marginal likelihood of $w_{dn}$ to optimize the parameters. As we do not know the topic proportion $\theta_{d}$ and topic assignment $z_{dn}$ in the generative process, we have to marginalize both latent variables. We first marginalize the topic proportion $\theta_{d}$, so the log likelihood $p\left(w_{dn}|\alpha^{t},\rho \right)$ is defined as


(6) pwdnαt,ρ=∫pθdpwdnθd,αt,ρdθd.

We then marginalize topic assignment $z_{dn}$ to compute the conditional distribution $p(w_{dn}|\theta_{d},\alpha^{t},\rho )$:


(7) pwdnθd,αt,ρ=∑k=1Kpzdn=kpwdnβzdnt.

After getting the log likelihood for each word, we then get the log likelihood loss function over parameter $\alpha^{t}$ and ρ:


(8) Llkα,ρ=∑t=1T∑d∈Dt∑w∈dlog⁡pwαt,ρ.

We use amortized variational inference to approximate the posterior distribution of topic proportion $\theta_{d}$ for document d (Kingma and Welling 2013). Particularly, we use neural networks μ and θ that take document d as input to predict the mean and variance of a Gaussian distribution. This Gaussian distribution is then used as the approximated posterior distribution of $\theta_{d}$. Formally, where ν denotes the parameters of the inference neural networks. We leveraged a recurrent neural network as the inference model *q* in our implementation. This approximate distribution can be leveraged to compute the evidence lower bound (ELBO) of the marginal log likelihood. ELBO is a function of the generative model parameters α,ρ and the variational parameters ν:


(9) qνθdd=LNμνD,σνD,

(10) LELBOα,ρ,ν=∑t=1T∑d∈Dt(∑w∈dEqlog⁡pwαt,ρ.-KL(qν|pθd))

We then optimize $L_{\mathrm{ELBO}}$ with regard to parameters (α,ρ,ν) using minibatch Monte Carlo approximation.

### 2.5 Topic diversity loss

Inspired by the multitask learning method, we optimize two additional loss terms mentioned in this section and Section 2.6 (Ruder 2017). We propose a TD loss to make extracted topics more informative. This loss encourages each topic representation to be far away from each other in the training process. Formally, where $\mathrm{Dis}(x_{1},x_{2})$ can be any distance metric. Specifically, we use Euclidean distance in our model.


(11) LTD=∑t=1T∑1≤i,j≤kDisαit,αjt,

### 2.6 IC classification loss

We propose an IC classification loss to let inferenced topic proportions of each document contain information for IC prediction. In the training stage, a fully connected neural network F(x) takes the inferenced topic proportion $\theta_{d}$ as the input and outputs a probability for each IC regarding which grant document might belong to it: where CE represents the cross-entropy loss. We then calculate the final loss function by adding up all three losses:


(12) LIC=∑t=1T∑d∈DtCEFθd,yd,

(13) Lα,ρ,ν=LELBO+λ1LTD+λ2LIC.

We optimize this loss function with gradient descent to compute the optimal topic representations α, word embeddings ρ, and variational parameters ν.

### 2.7 Evaluation methods

We expect a good topic model to generate topics that are interpretable and informative. Moreover, these topics should be capable of reconstructing the original word distribution. Therefore, we evaluate the performance of our topic model using metrics including TC, TD, and test perplexity (Rosen-Zvi *et al.* 2004, Mimno *et al.* 2011).

TC measures the similarity of words drawn from a topic, indicating whether the topic is semantically interpretable. Formally, we compute TC for a topic by selecting the top-*p* words from the topic and averaging over the similarity between any pair of words: where $w_{i},w_{j}$ are drawn from the top-*p* words of a topic and f is a similarity measure. In this article, we choose three different functions for f: pairwise comparison based on context window (CA), Fitelson’s confirmation measure (CP), and normalized pointwise mutual information (NPMI) (Aletras and Stevenson 2013, Röder *et al.* 2015).


(14) TC=1p2∑1≤i,j≤pfwi,wj,

TD penalizes the repetitive or similar topics by calculating the repetitions of topic words. We use the proportion of unique top-*p* words in topics to compute TD in our article. Formally, where K is the number of topics and $N_{u}$ is the number of unique words.


(15) TD=NuK×p,

Perplexity measures the likelihood of a topic model on a held-out test dataset.

### 2.8 Experimental settings

We utilize BioWordVec as the word embeddings for our method (Zhang *et al.* 2019). BioWordVec encompasses 200-dimensional word embeddings trained on biomedical text with a biomedical controlled vocabulary, which are more suitable to NIH grant abstract text. Note that the parameters of the word embedding layer were also updated during the training process.

We use 85% of the *Grant* dataset for training, 5% for validation, and 10% for testing. For the purpose of topic quality evaluation and trend analysis, we trained *Turtling* with a topic number of *K* = 50. We set the learning rate of *Turtling* to be 0.001 with a small weight decay. We set the batch size to be 1024 and the dropout rate to be 0.1. We set the hyperparameters $\lambda_{1}$ and $\lambda_{2}$ in Equation (13) to be 1 and 0.5. We set the hyperparameters ϵ,δ, and γ in Equations (3), (4), and (5) to be 0.01. We trained our model for 500 epochs on an Nvidia RTX 3090 GPU. We tested different choices of hyperparameters K,ϵ,δ, and γ to select the best value above. Results for hyperparameters tuning are shown in Supplementary Fig. S1.

In Section 3.4, we leveraged *Turtling* for IC classification. Specifically, we leveraged the topic proportion vector as the input feature to a random forest classifier, which is lighter and more interpretable compared to models using entire documents as input. For a fair comparison, we applied the PCA method to the BOW representation of each document with the same output dimension as the number of topics. We also trained a DETM model and extracted topic proportions as input features. Here, we selected 20 as the number of topics. As sometimes, we expected the model to predict several possible IC selections, we computed the top-5 accuracy as well as the top-1 accuracy. We also tested the performance of a neural network classifier instead of a random forest classifier and the results are shown in Supplementary Fig. S2.

## 3 Results

Here, we applied *Turtling* on the *Grant* dataset and evaluated its performance on the extracted topic quality and IC classification accuracy, as discussed in the following sections. In Section 3.1, we evaluate the performance of our model and compare it with baseline methods on several topic quality metrics, demonstrating that *Turtling* improves the quality of extracted topics. In Section 3.2, we leverage the topics extracted by *Turtling* from the *Grant* dataset to analyze the research topic trend in recent years. In Section 3.3, we create a topic heatmap and the topic hierarchy to intuitively show the correlation between extracted topics. In Section 3.4, we use the topic proportions as an input feature to predict IC labels on the test dataset, indicating that topics extracted by *Turtling* are strongly correlated with the selection of NIH institutes.

### 3.1 *Turtling* improves topic quality from NIH grant text

We applied *Turtling* on the *Grant* dataset and benchmarked its performance from three different aspects. First, we compared the baseline model DETM (Dieng *et al.* 2019) and our model using TC (CA, CP, and NPMI), TD and tested perplexity described in detail in Section 2.7. We also evaluated an ETM model on 1 year of data without time information (Dieng *et al.* 2020). As shown in Table 2, *Turtling* outperformed DETM on all metrics, especially in TD and CP. Furthermore, *Turtling* achieved comparable topic quality results with the static topic modeling method ETM. Note that ETM was evaluated on a single-year dataset which is much smaller than the complete dataset than the other two methods used, as ETM cannot capture the dynamic evolution of topics. We also compared *Turtling* with a nongenerative topic modeling method, BERTopic (Grootendorst 2022). Results are shown in Supplementary Table S1 and *Turtling* also achieved competitive results on TC and TD.

**Table 2.** Topic quality results^a^.

Method	CA	CP	NPMI	TD	Perplexity
ETM	**0.13**	**0.17**	0.015	0.82	**2986.8**
DETM	0.10	−0.2	0	0.52	3617.9
*Turtling*	0.11	0.15	**0.023**	**0.86**	3120.7

a We compared the performance of our model with several baseline topic models on TC and topic divergence. Bold indicates ETM model outperformed in metrics CA, CP and Perplexity, whereas DETM model excelled in metrics NPMI and TD.

### 3.2 *Turtling* highlights dynamic research topic changes over the past decades

**Figure 3.**
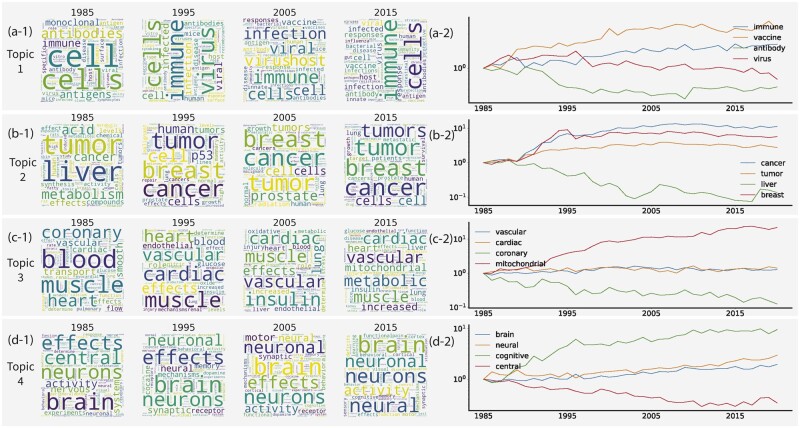
Wordcloud trend and keywords proportion trend for four topics across decades. For each topic, we selected four keywords and normalized their generative probability for each keyword. We then plot the normalized probability in each year from 1985 to 2020. We also select four specific years to create the wordcloud according to the generative probability of each topic.

As shown in the right part of Fig. 3, we visualized the generative probability for some words with high generative probability in four example topics from 1985 to 2020. Note that in this plot, we normalized the generative probability for each keyword by setting the generative probability of this word in 1985 as 1 so that we can focus on the developing trend for each keyword across different years.

First, we observed clear trends of research topic and word distribution across years from our *Turtling* results. For instance, “immune” and “vaccine” (Topic 1) related research has been increasingly attracting research attention within Topic 1 since 1985 as shown in Fig. 3a-2. Furthermore, within Topic 2, breast cancer is one of the top increasing words, indicating significantly expanded funding opportunities in the past 20 years under this topic, as shown in Fig. 3b-2. Similarly, mitochondrial and brain-related also research topics demonstrated a noticeable popularity gain in recent years. We further show the evolutionary trend of each topic of a 20-topic *Turtling* model in Supplementary Fig. S3.

Next, we showed the temporal evolution of example words for biomedical research topics. For each of the most popular topics mentioned above, we listed some examples of top words in 1985, 1995, 2005, and 2015. To intuitively show the distribution of each word, we generated wordcloud for each topic at different time points. In wordcloud plots, larger fonts of words represent a higher generative probability of that word. The visualization results are shown in the left part of Fig. 3.

Furthermore, we observed the keywords for each topic from the wordcloud across years. In 1985, “blood” was a major concern in Topic 3 which contains vascular-related research, but “cardiac” had been more popular since 1995. We also inferred the main topic name for each plot according to the top words in that topic. For example, given “antibody,” “vaccine,” and “virus” in Fig. 3a-1, we can infer that the research field for this topic is likely to be “immune.”

### 3.3 *Turtling* extracts hierarchy research topic relationships from grant text

**Figure 4.**
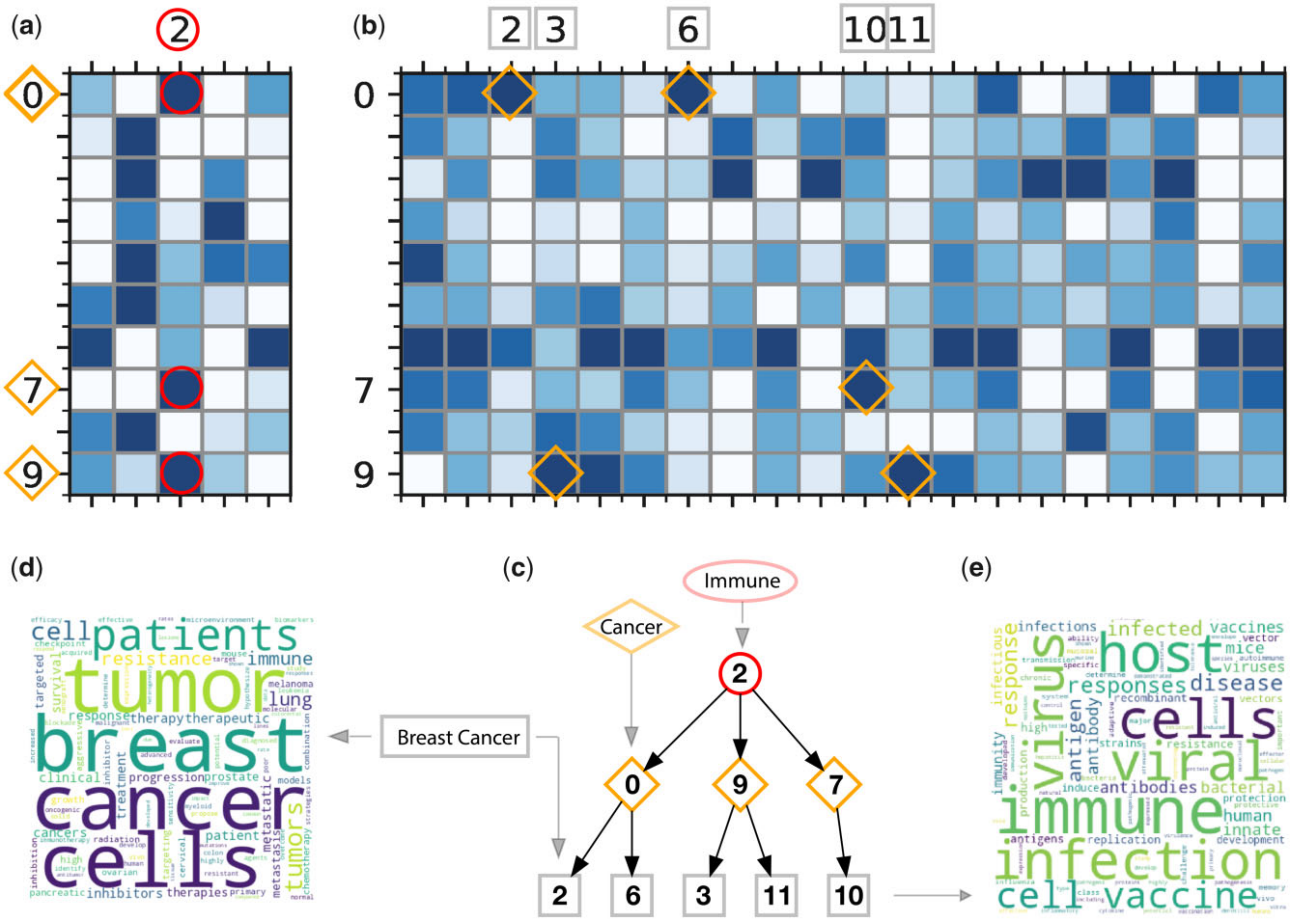
Heatmap and hierarchy trees for grant topics. We trained *Turtling* with 5, 10, and 20 topics, and calculated the correlation factors between different topics. We show heatmaps of correlation between a 5-topic model and a 10-topic model (a), and a 10-topic model and a 20-topic model (b). We further created the hierarchy trees for these topics in (c), and extracted the word logos using the word frequencies in topic 2 (d) and topic 10 (e).

Next, we aim to explore the subfields of extracted research topics by examining connections of models trained with different topic numbers. As shown in Fig. 4, we trained *Turtling* models with 5, 10, and 20 topics on the same collected grant text data. As a result, topics in the 5-topic model can be interpreted as broad research areas, while the subfields can be represented by topics in the 10- and 20-topic models. Consequently, the broad research area and subfield connections can be directly measured by the similarities of topic embeddings from different models.

We found that Topic 2 in the 5-topic model is highly enriched in “immune” terminologies (the circle with number 2 in Fig. 4a and b). We explored its most closely associated subfields by calculating its most closely relevant topics in the subsequent 10 and 20-topic models, as shown in the heatmaps (Fig. 4a and b). For instance, Topics 0, 7, and 9 in the 10-topic model showed the highest correlation with Topic 2 in the 5-topic model. We can further trace down the higher resolution subfields in the 20 topic models by showing that Topics 2 and 6, 3 and 11, and Topic 10 are most connected to our subtopics in 10 topic models. We further extracted the word logo using the word frequencies in each topic and found that cancer and viral infection are important subfields for the “immune” topic we selected (Fig. 4c). These results demonstrate that *Turtling’*s ability to extract hierarchical relationships between different research fields in a completely data-driven manner.

### 3.4 *Turtling* improves IC classification accuracy

Besides traditional research topic extraction tasks, an ideal grant analysis model should be able to accurately predict the funding IC and provide appropriate suggestions for future grant text data. Therefore, we further tested *Turtling’s* performance on an IC classification task using the topic distributions (details in Section 2.8).

**Figure 5.**
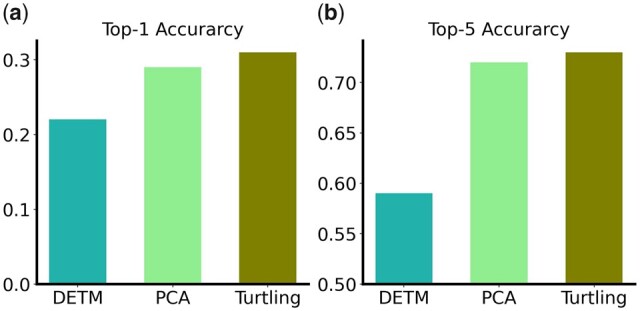
IC classification accuracy. We compared top-1 (a) and top-5 (b) accuracy of IC classification task using DETM, PCA, and *Turtling*.

We benchmarked with traditional PCA and DETM models using top-1 and top-5 IC assignments. As shown in Fig. 5, *Turtling* achieved a 31.6% top-1 accuracy, significantly higher than results from DETM and PCA (22.3% and 29.1% top-1 accuracy, respectively). Furthermore, *Turtling* achieved a 73.8% top-5 accuracy which outperforms results from both methods (59.2% and 72.3% top-5 accuracy, respectively). These experimental results showed that our method outperformed both of the baseline methods, demonstrating the effectiveness of using topic proportions generated by our model for IC classification.

### 3.5 *Turtling* separates documents from different ICs

**Figure 6.**
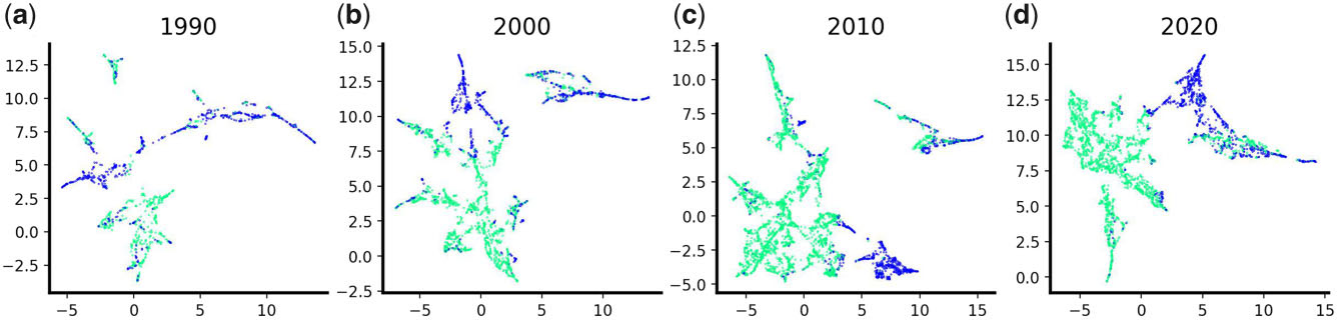
UMAP visualization of topic proportions. We leveraged UMAP to reduce the dimension of topic proportion vector for each document. The documents come from “NCI” and “NIMH” in years 1990 (a), 2000 (b), 2010 (c) and 2020 (d), and they are represented by two different colors, respectively.

To intuitively demonstrate topic proportion vectors generated by *Turtling* are separable among different ICs, we then visualized the vector of grant documents from two ICs in 1990, 2000, 2010, and 2020. We selected grants from the “National Cancer Institute” (NCI) and the “National Institute of Mental Health” (NIMH), as we expect the topics to vary significantly between these two ICs. We used UMAP to generate a two-dimensional representation of topic proportion vectors for visualization (Mcinnes 2018). The results are shown in Fig. 6. Each dot with a certain color represents a document from a specific IC. We can observe from the plots that data points with different colors tend to form different clusters, indicating that each IC has its own topic preference.

To sum up, qualitative and quantitative analysis both show that the topic proportions generated by *Turtling* provide a useful and interpretable way for IC prediction tasks.

## 4 Discussion

In this article, we developed *Turtling*, a time-aware topic model to analyze documents from a large grant corpus funded by the NIH. We constructed the *Grant* dataset, which contains 466 730 grant abstract documents and their corresponding ICs over the past 36 years. *Turtling* is novel with three main characteristics: the combination of biomedical word embedding and topic modeling, the time-aware nature of the graphical model, and the multitask loss which includes topic divergence loss and IC classification loss.

We trained our model by optimizing the traditional ELBO as well as the TD loss and the IC classification loss. Experimental results showed our method outperformed baseline methods on all of the metrics. We then leveraged *Turtling* to extract research topic trends from 1985 to 2020. We further demonstrated that the topic proportions generated by our method can be used for IC prediction.

In the future, we expect several extensions could be easily incorporated into our method for further performance improvement. First, *Turtling* leveraged a naïve random forest classifier for IC classification, which could be substituted with more advanced deep classification models like transformers (Vaswani *et al.* 2017). Second, pretrained language models (PLMs) have become popular in many NLP applications (Peters *et al.* 2018, Devlin *et al.* 2019). Previous works have applied large PLMs to topic modeling tasks, but none of them considered the time-aware topic modeling scenario (Zhang et al. 2022). As PLMs trained on biomedical text would contain large amounts of biomedical domain information, it may further improve the performance of topic models on the *Grant* dataset (Lee *et al.* 2020). Lastly, the training process of *Turtling* is time-consuming due to its sequential inference strategy, posing a potential need for efficient inference and sampling methods.

We have implemented *Turtling* as an open-source software that is freely downloadable to the public. With the exponential growth of publicly available grant text data, *Turtling* can be a valuable tool for investigators and funding agencies to gain research insights in a completely data-driven manner.

## Supplementary Material

vbad096_Supplementary_Data

## Data Availability

All the data from this work has been downloaded directly from NIH reporter.

## References

[vbad096-B1] Aletras N , Stevenson M. Evaluating topic coherence using distributional semantics. *Proceedings of the 10th International Conference on Computational Semantics (IWCS 2013) – Long Papers*. Potsdam, Germany, Association for Computational Linguistics, 2013, 13–22.

[vbad096-B2] Bird S , Loper E. NLTK. In: *Proceedings of the ACL 2004 on Interactive Poster and Demonstration Sessions*. Morristown, NJ: Association for Computational Linguistics, 2004.

[vbad096-B3] Blei DM, Ng A, Jordan M. Latent Dirichlet allocation. J Mach Learn Res 2003;3:993–1022.

[vbad096-B4] Blei DM , Lafferty JD. A correlated topic model of science. Ann Appl Stat 2007;1:17–35.

[vbad096-B5] Blei DM , Lafferty JD. Dynamic topic models. In: *Proceedings of the 23rd International Conference on Machine Learning, ICML ’06*. New York, NY: Association for Computing Machinery, 2006, 113–120.

[vbad096-B6] Devlin J , Chang M-W, Lee K, Toutanova K. BERT: pre-training of deep bidirectional transformers for language understanding. *Proceedings of the 2019 Conference of the North American Chapter of the Association for Computational Linguistics: Human Language Technologies*, Volume 1, 2019.

[vbad096-B7] Dieng AB , Ruiz FJR, Blei DM. The dynamic embedded topic model. arXiv preprint arXiv:1907.05545, 2019, preprint: not peer reviewed.

[vbad096-B8] Dieng AB , Ruiz FJR, Blei DM. Topic modeling in embedding spaces. Trans Assoc Comput Linguist 2020;8:439–53.

[vbad096-B9] Grootendorst M. BERTopic: neural topic modeling with a class-based TF-IDF procedure. arXiv preprint arXiv:2203.05794 , 2022, preprint: not peer reviewed.

[vbad096-B10] Kingma DP , Welling M. Auto-encoding variational Bayes. In: *ICLR 2014*, Banff, AB, Canada, 2014.

[vbad096-B11] Lee J , Yoon W, Kim S et al BioBERT: a pre-trained biomedical language representation model for biomedical text mining. Bioinformatics 2020;36:1234–40. 31501885 10.1093/bioinformatics/btz682 PMC7703786

[vbad096-B12] McInnes L , Healy J, Saul N et al UMAP: uniform manifold approximation and projection. J Open Source Softw 2018;3:861.

[vbad096-B13] Mikolov T , Chen K, Corrado GS, Dean J. Efficient estimation of word representations in vector space. ICLR 2013, Scottsdale, AZ, 2013a.

[vbad096-B14] Mikolov T , Sutskever I, Chen K et al Distributed representations of words and phrases and their compositionality. In: Burges CJ (ed.), Advances in Neural Information Processing Systems. Lake Tahoe, NV: Curran Associates, Inc., 2013b, 3111–9.

[vbad096-B15] Mimno D, Wallach HN, Talley E et al Optimizing semantic coherence in topic models. In: *Proceedings of the 2011 Conference on Empirical Methods in Natural Language Processing*. Edinburgh, UK: Association for Computational Linguistics, 2011, 262–272.

[vbad096-B16] Park J, Blume-Kohout M, Krestel R et al Analyzing NIH funding patterns over time with statistical text analysis. In: *Workshops at the Thirtieth AAAI Conference on Artificial Intelligence*, 2016.

[vbad096-B17] Peters M, Neumann M, Iyyer M et al Deep contextualized word representations. In: *Proceedings of the 2018 Conference of the North American Chapter of the Association for Computational Linguistics: Human Language Technologies, Volume 1 (Long Papers)*. Stroudsburg, PA: Association for Computational Linguistics, 2018.

[vbad096-B18] Röder M, Both A, Hinneburg A. Exploring the space of topic coherence measures. In: *Proceedings of the Eighth ACM International Conference on Web Search and Data Mining, WSDM ’15*. New York, NY: Association for Computing Machinery, 2015, 399–408.

[vbad096-B19] Rosen-Zvi M, Griffiths T, Steyvers M, Smyth P. The author-topic model for authors and documents. In: *Proceedings of the 20th Conference on Uncertainty in Artificial Intelligence, UAI ’04*. Arlington, VA: AUAI Press, 2004.

[vbad096-B20] Ruder S. An overview of multi-task learning in deep neural networks. arXiv preprint arXiv:1706.05098, 2017, preprint: not peer reviewed.

[vbad096-B21] Talley EM , Newman D, Mimno D et al Database of NIH grants using machine-learned categories and graphical clustering. Nat Methods 2011;8:443–4. 21623347 10.1038/nmeth.1619 PMC5361216

[vbad096-B22] Vaswani A, Shazeer N, Parmar N et al Attention is all you need. In: Guyon I (ed.), Advances in Neural Information Processing Systems. Long Beach, CA: Curran Associates, Inc., 2017, 6000–10.

[vbad096-B23] Zhang L, Hu X, Wang B et al Pre-training and fine-tuning neural topic model: A simple yet effective approach to incorporating external knowledge. In: *Proceedings of the 60th Annual Meeting of the Association for Computational Linguistics (Volume 1: Long Papers).* Stroudsburg, PA: Association for Computational Linguistics, 2022. 10.18653/v1/2024.acl-long.199 PMC12007664 40255468

[vbad096-B24] Zhang Y , Chen Q, Yang Z et al BioWordVec, improving biomedical word embeddings with subword information and MeSH. Sci Data 2019;6:52. 31076572 10.1038/s41597-019-0055-0 PMC6510737

