# Supplementary material for: Turtling: a time-aware neural topic model on NIH grant data
Source: Bioinform Adv. 2023 Jul 24;3(1):vbad096. doi: 10.1093/bioadv/vbad096 (PMC11216609; doi:10.1093/bioadv/vbad096)
Supplement: vbad096_Supplementary_Data [file vbad096_supplementary_data.pdf]

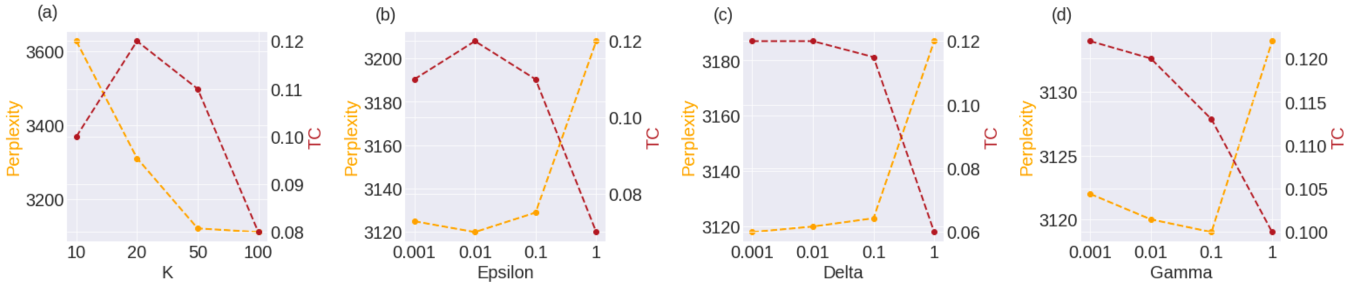


**Supplementary Figure 1.** Performance of Turtling using different selection of hyperparameters $K, \epsilon, \delta$ and $\gamma$.

| Metric | CA | CP | NPMI | TD |
|---|---|---|---|---|
| BERTopic | 0.11 | -0.08 | 0.02 | 0.93 |
| Turtling | 0.11 | 0.15 | 0.023 | 0.86 |

**Supplementary Table 1.** Performance comparison between Turtling and BERTopic.

| Method | Top-10 words in topic 1 |
|---|---|
| BERTopic | unreadable, health, thereby, public, mental, care, improving, quality, community, medical. |
| Turtling | tumor, liver, metabolism, acid, cancer, effects, compounds, synthesis, breast, lung. |

**Supplementary Table 2.** Top-10 words in topic 1 for BERTopic and Turtling.

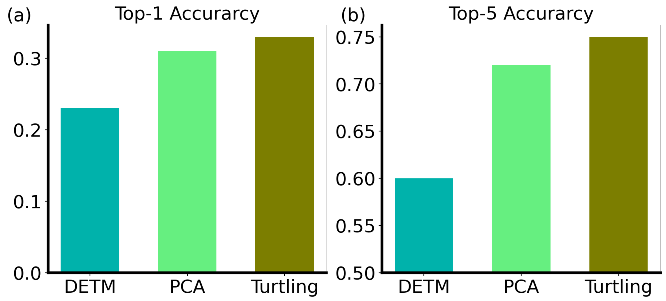


**Supplementary Figure 2.** IC prediction accuracy of a neural network classifier using vector representations generated from DETM, PCA and Turtling.

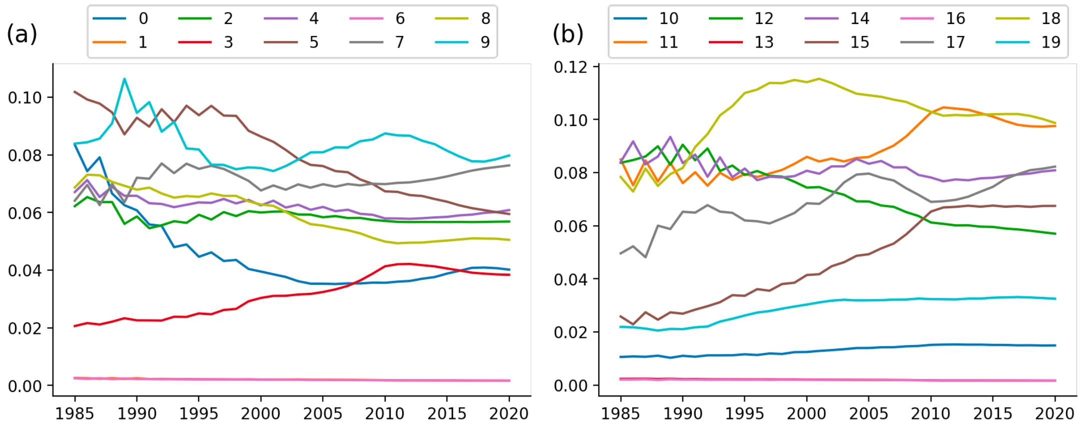


**Supplementary Figure 3.** Topic proportion evolution trend of a 20-topic Turtling model across 35 years.
